# Serum VEGF-A and CCL5 levels as candidate biomarkers for efficacy and toxicity of regorafenib in patients with metastatic colorectal cancer

**DOI:** 10.18632/oncotarget.9187

**Published:** 2016-05-05

**Authors:** Mitsukuni Suenaga, Tetsuo Mashima, Naomi Kawata, Takeru Wakatsuki, Yuki Horiike, Satoshi Matsusaka, Shingo Dan, Eiji Shinozaki, Hiroyuki Seimiya, Nobuyuki Mizunuma, Kensei Yamaguchi, Toshiharu Yamaguchi

**Affiliations:** ^1^ Department of Gastroenterological and Chemotherapy Cancer Institute Hospital of Japanese Foundation for Cancer Research, Koto-ku, Tokyo 135–8550, Japan; ^2^ Division of Molecular Biotherapy, Cancer Chemotherapy Center, Japanese Foundation for Cancer Research, Koto-ku, Tokyo 135–8550, Japan; ^3^ Division of Molecular Pharmacology, Cancer Chemotherapy Center, Japanese Foundation for Cancer Research, Koto-ku, Tokyo 135–8550, Japan; ^4^ Department of Gastroenterological and Surgery Cancer Institute Hospital of Japanese Foundation for Cancer Research, Koto-ku, Tokyo 135–8550, Japan

**Keywords:** regorafenib, metastatic colorectal cancer, VEGF-A, CCL5, prognostic marker

## Abstract

Regorafenib is an oral multi-kinase inhibitor used as salvage therapy for metastatic colorectal cancer (mCRC). We tested whether serum cytokine levels are associated with clinical outcome in the mCRC patients receiving regorafenib. Serum samples were collected before treatment start, day 21, and progressive disease, and eleven angiogenic and inflammatory cytokine serum levels were examined. Fifty-four patients of a total of 62 enrolled patients were eligible for the analyses. The chemokine ligand 5 (CCL5) levels ≤ cut-off value (59959 pg/ml) at baseline was associated with relative tumor shrinkage (*P* = 0.021), better progression-free survival (PFS) (*P* = 0.036) and overall survival (OS) (*P* = 0.019). Vascular endothelial growth factor A (VEGF-A) levels showing a decrease on day 21 were significantly associated with a better PFS (*P* = 0.021). CCL5 levels ≤ cut-off was associated with any grade hand-foot skin reaction (HFSR) (*P* = 0.025) and thrombocytopenia (*P* = 0.013). Low chemokine ligand 2 levels at baseline were associated with grade 2 ≤ HFSR. High angiopoietin-2 and basic fibroblast growth factor (bFGF) levels at baseline were associated with grade 3 ≤ total bilirubin increase and transaminases increase, respectively. Low bFGF levels at baseline were associated with grade 3 ≤ hypertension. No correlation with severe events was observed. Baseline serum CCL5 levels and decrease of the serum VEGF-A levels may serve as potential predictive markers for survival or treatment-specific toxicities in mCRC patients receiving regorafenib.

## INTRODUCTION

Colorectal cancer is the leading cause of cancer death among women and third highest cause of cancer death among men in Japan [1]. Oxaliplatin (L-OHP), irinotecan (CPT-11), fluorouracil (5-FU), and several molecular-targeting agents are key drugs in the treatment of metastatic colorectal cancer (mCRC) [2–7]. Most patients receive regimens that include a combination of these key drugs, and cetuximab or panitumumab are applied if the tumor has wild-type *KRAS* as well as *NRAS* [8–12]. Investigating which biologic agents were appropriate in the first-line treatment between bevacizumab and anti-epidermal growth factor receptor (EGFR) agents has been focused in two phase III trials, resulting in similar benefit on survival [13, 14]. The results showed the sequential use of the established regimens containing these key agents prolong overall survival (OS) by up to 30 months [13, 14]. Lately, novel oral agents such as regorafenib [15] and TAS-102 [16, 17] have been approved as salvage therapy for mCRC.

Regorafenib is an oral multi-kinase inhibitor that blocks the activity of several protein kinases involved in the multiple biological processes for progression and development of cancer; these kinases include vascular endothelial growth factor receptor (VEGFR) 1–3 and tyrosine kinase with immunoglobulin and epidermal growth factor homology domain 2 (TIE2) involved in tumor angiogenesis, V-kit Hardy-Zuckerman 4 feline sarcoma viral oncogene homolog (KIT), rearranged during transcription tyrosine kinase (RET), rat fibroblastoma 1 (RAF1), and v-raf murine sarcoma viral oncogene homolog B1(BRAF) in oncogenesis, and platelet derived growth factor receptor (PDGFR) and fibroblast growth factor receptor (FGFR) in the tumor microenvironment [15]. Treatment with regorafenib has shown significant benefits for OS and progression-free survival (PFS) in patients with previously treated metastatic colorectal cancer in two placebo-controlled phase 3 trials, CORRECT [16] and CONCUR [18], which compared regorafenib to placebo. To investigate the effect of tumor mutation status, plasma DNA concentration, or plasma protein concentration on treatment outcomes and response to treatment in patients treated with regorafenib, a retrospective exploratory analysis was performed for the clinical activity of regorafenib in biomarker subgroups of the study population of CORRECT [19]. Although the results conclusively propose Beads, Emulsion, Amplification, Magnetics (BEAMing) analysis of circulating DNA as a noninvasive viable approach to obtain real-time tumor-associated genotypic information, there have not yet been useful predictive biomarkers of regorafenib as a convenient method for clinical practice before treatment or early phase of treatment start.

Furthermore, remarkable differences in adverse event profiles were observed between Japanese and non-Japanese subpopulations such as hand-foot skin reaction (HFSR), hypertension or anorexia [20]. Treatment-related liver dysfunction was also more frequent in Japanese subpopulation than non-Japanese subpopulation. Thereby, frequent and careful monitoring of liver function is strongly recommended, especially within the first two cycles of the treatment.

Cytokine levels as well as their changes in liquid biopsy samples could potentially be useful to monitor or predict disease progress and treatment outcome. Previously, it was reported that colon cancer overexpresses VEGF-A and serum VEGF-A with higher levels in colon cancer patients compared with normal population, especially in advanced tumors [21]. Our gene expression analysis also revealed that treatments with molecularly targeted anti-cancer agents significantly changed the expression of cytokines [22] (http://scads.jfcr.or.jp/db/cs/). In the analysis, we observed that the regorafenib treatment significantly changed the expression of secreting factors such as VEGF-A (http://scads.jfcr.or.jp/db/cs/files/C099-up2-100.csv). These observations suggest that serum cytokine levels would be changeable in patients during treatment with regorafenib and the changes could serve as specific markers for sensitivity to the agent. Here, we analyzed changes of soluble cytokines in the serum after regorafenib treatment, including angiogenic factors as well as inflammatory factors that are involved with cancer progress.

## RESULTS

### Baseline patient characteristics

The 62 eligible patients were enrolled between March 2013 and December 2014, but 8 patients were excluded because of no treatment or defect of sample at any blood collecting point. Finally, 54 patients were included in the analysis of the efficacy and safety. Their characteristics were as follows: median age of 65 years (range: 34–78 years); ECOG PS of 0 in 63.0%, and prior chemotherapy regimen of ≥ 3 in 74.1%. All patients received 5-FU, L-OHP and CPT-11, and most of the patients also received molecular-targeting agents. The details are shown in Table 1. The median follow-up period was 17.7 months. The median number of treatment cycles was 3 (range: 1–20).

**Table 1:** Baseline characteristics of the patients (*n* = 54)

Characteristic	*N*	%
Sex		
Male Female	29 25	53.7 46.3
Age		
Median Range	65 34–78	
ECOG PS		
0 1	34 20	63.0 37.0
Site of primary tumor		
Colon Rectum	38 16	70.4 29.6
Primary tumor		
Resected Unresected	45 9	83.3 16.7
Histology		
Well Moderately Poorly Unknown	16 33 3 2	29.6 61.1 5.6 3.7
Adjuvant treatment		
Yes No	19 35	35.2 64.8
KRAS status		
Wild-type Mutant Unknown	36 17 1	66.7 31.5 1.9
Sites of metastasis		
Liver Lung Lymph nodes Peritoneum Other	34 29 29 12 10	63.0 53.7 53.7 22.2 18.5
Number of metastatic sites		
1 ≥ 2	19 35	35.2 64.8
Previous treatment lines		
1 2 3 4	1 13 35 5	1.9 24.1 64.8 9.3
Exposure to chemotherapeutic agents		
5-FU L-OHP CPT-11 Bevacizumab Anti-EGFR (CET or PAN)	54 54 54 50 38	100 100 100 92.6 70.4
Median serum level at baseline (range)		
CEA, ng/ml CA19-9, U/ml	44.0 (2.0–5664.4) 56.6 (2.0–32352.9)	

ECOG PS, Eastern Cooperative Oncology Group performance status; 5-FU, 5-fluorouracil; L-OHP, oxaliplatin; CPT-11, irinotecan; EGFR, epidermal growth factor receptor; CET, cetuximab; PAN, panitumumab; CEA, carcinoembryonic antigen; CA19-9, carbohydrate antigen 19-9.

### Efficacy

**Figure 1:**
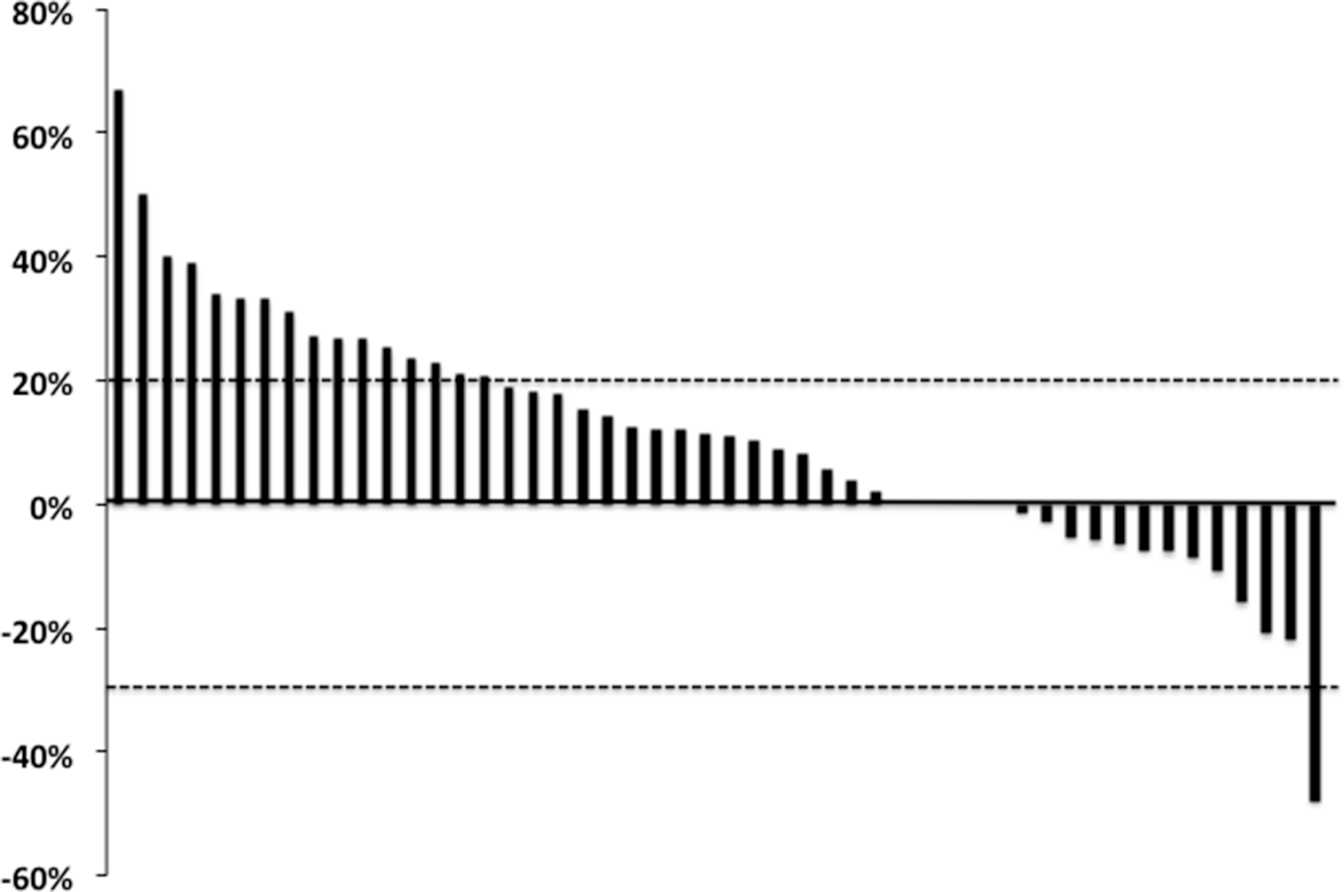
Waterfall plot of the best tumor response in patients with metastatic colorectal cancer receiving regorafenib Relative tumor shrinkage was observed in 18/54 patients (33.3%).

The tumor response was assessed in 52 of the 54 patients. It was not evaluable in 2 patients due to adverse events that developed before the end of the first treatment cycle. The objective response rate (ORR) was 1.9% (95% confidence interval [CI]: −1.9%–5.6%), and disease control was achieved in 51.9% (95% CI: 38.1–65.6%) of the patients after 8 weeks of treatment. A waterfall plot of the tumor response after 8 weeks in the evaluable 50 patients is shown in Figure 1. Two patients were excluded from this analysis because their target lesions became unmeasurable at the first evaluation, but were classified as having progressive disease with the non-target lesions or the appearance of new metastatic lesions. Relative tumor shrinkage (rTS) was observed in 33.3% of the enrolled patients. Median progression-free survival (PFS) was 3.2 months (95% CI: 2.1–4.3 months), while median OS was 8.7 months (95% CI: 5.7–11.7 months).

According to the results from the univariate analysis for clinical factors, PFS was significantly shorter in patients with liver metastases (hazard ratio [HR]: 3.27; 95% CI: 1.59–6.69, *P* = 0.001) and patients with two or more metastatic sites (HR: 1.99; 95% CI: 1.04–3.78, *P* = 0.036), while there was no significant improvement of PFS in patients with a KRAS mutant tumor (HR: 0.53; 95% CI: 0.27–1.03, *P* = 0.062). OS was significantly shorter with cases of liver metastasis (HR: 2.90; 95% CI: 1.43–5.88, *P* = 0.003) and lymph node metastasis (HR: 2.04, 95% CI: 1.06–3.94, *P* = 0.033), while primary tumor location was not associated with OS (HR: 0.51, 95% CI: 0.89–4.65, *P* = 0.092).

### Serum cytokine levels at each test point and efficacy

**Figure 2:**
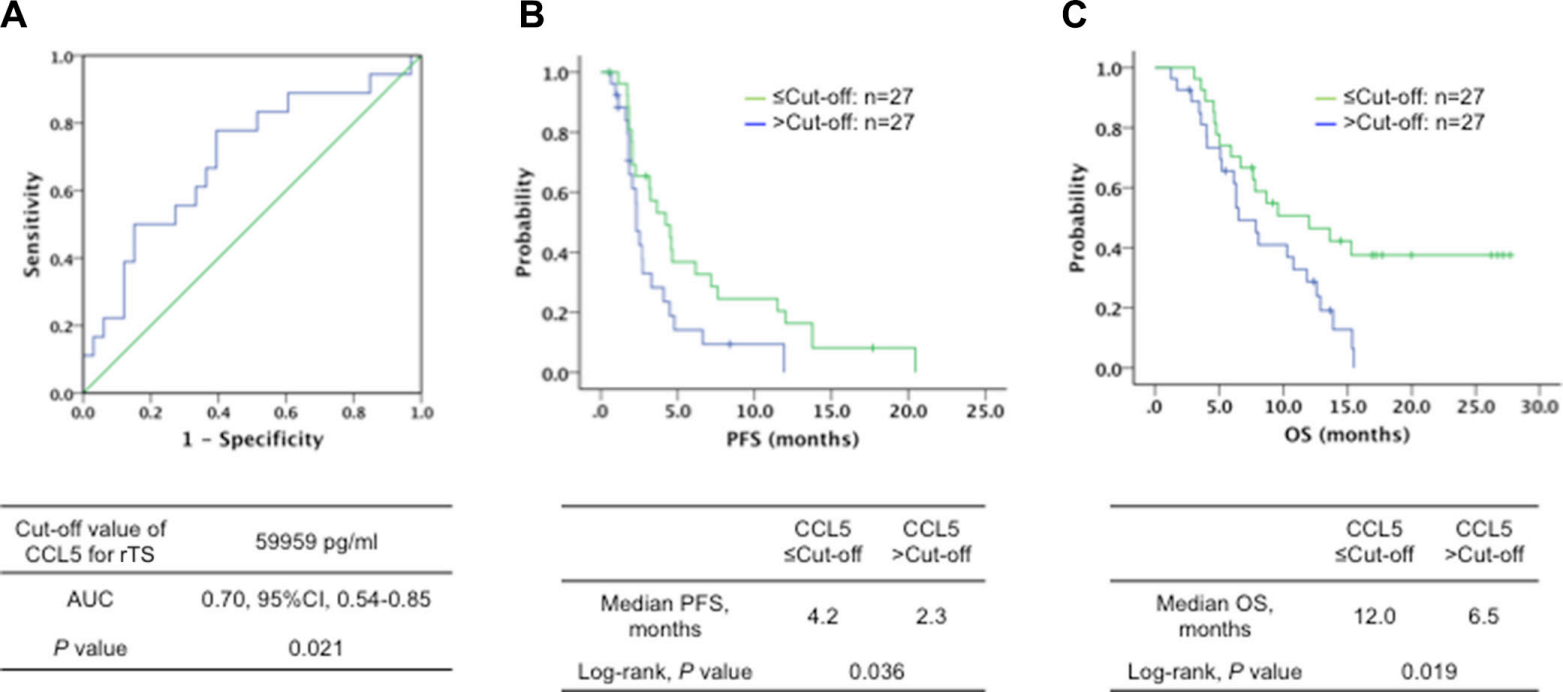
(**A**) Receiver operating characteristic curve (ROC) with area under the curve (AUC) (0.70, 95% CI, 0.54–0.85, *P* = 0.021) for detecting relative tumor shrinkage (rTS) by serum CCL5 levels, (**B**) progression-free survival (PFS) (4.2 vs. 2.3 months, Log-rank test, *P* = 0.036) and (**C**) overall survival (OS) (12.0 vs. 6.5 months, Log-rank test, *P* = 0.019) in patients with metastatic colorectal cancer treated with regorafenib according to the baseline serum CCL5 levels ≤ (—, *n* = 27) or > (—, *n* = 27) the cut-off value (determined by ROC curve analysis).

We measured eleven angiogenic and inflammatory cytokine levels in the serum of all 54 eligible patients (Table 2). The Student's unpaired *t* test showed that the serum levels of CCL5 at baseline were significantly lower in the subgroup with tumor shrinkage (50500 ± 23800 vs. 65800 ± 23200 pg/ml, *P* = 0.030), while a trend was similar on day 21 after treatment start. No significant differences were observed in other cytokines in respect to both disease control (DC) and rTS (Table 2). According to the ROC analysis (Figure 2A), rTS was highly observed in patients with serum levels of CCL5 at baseline less than the cut-off point of 59959 pg/ml (*P* = 0.021). There was also significant improvement in PFS (4.2 vs. 2.3 months, *P* = 0.036) and OS (12.0 vs. 6.5 months, *P* = 0.019) in patients with baseline CCL5 low levels (≤ cut-off value) (Figure 2B, 2C). There was no remarkable correlation between other cytokines with respect to PFS or OS. In one-way ANOVA for three measurement points (at baseline, day 21 after treatment start, and at the time of confirmed progressive disease), significant differences in the mean of serum cytokine levels of VEGF-C (*P* < 0.001), SDF-1 (*P* = 0.040), bFGF (*P* = 0.004), PlGF (*P* < 0.001) and CCL2 (*P* = 0.015) were shown in total patients.

**Table 2:** Tumor response and serum cytokine levels (*n* = 54)

Cytokines and measurement points		DC*	Mean ± SD (pg/ml)	*P* value	rTS**	Mean ± SD (pg/ml)	*P* value
Ang-1	pre	N	31440 ± 17300	0.483	N	30680 ± 15960	0.549
		Y	26700 ± 9640		Y	26520 ± 10180	
	day 21	N	22880 ± 18500	0.929	N	22580 ± 16580	0.995
		Y	22220 ± 13100		Y	22520 ± 15060	
	PD	N	21280 ± 16420	0.383	N	21540 ± 14740	0.319
		Y	27180 ± 10960		Y	28440 ± 12260	
Ang-2	pre	N	2625 ± 1275	0.280	N	2685 ± 1205	0.241
		Y	3190 ± 2215		Y	3475 ± 2630	
	day 21	N	3660 ± 2555	0.371	N	3560 ± 2200	0.517
		Y	3125 ± 1675		Y	3155 ± 1980	
	PD	N	4500 ± 2685	0.755	N	4435 ± 2310	0.563
		Y	4870 ± 4815		Y	5570 ± 6465	
VEGF-A	pre	N	365 ± 340	0.993	N	325 ± 315	0.414
		Y	365 ± 465		Y	440 ± 550	
	day 21	N	565 ± 530	0.211	N	500 ± 475	0.507
		Y	405 ± 340		Y	415 ± 385	
	PD	N	370 ± 450	0.275	N	380 ± 420	0.078
		Y	535 ± 525		Y	675 ± 615	
VEGF-C	pre	N	4710 ± 2995	0.562	N	4570 ± 2645	0.813
		Y	4325 ± 1620		Y	4405 ± 1790	
	day 21	N	3405 ± 2120	0.792	N	3545 ± 1980	0.907
		Y	3545 ± 1685		Y	3480 ± 1715	
	PD	N	4230 ± 2455	0.764	N	4235 ± 2355	0.726
		Y	4425 ± 1795		Y	4490 ± 1510	
SDF-1	pre	N	3660 ± 1380	0.262	N	3640 ± 1360	0.125
		Y	3240 ± 1260		Y	3030 ± 1220	
	day 21	N	4250 ± 2120	0.384	N	4180 ± 2010	0.300
		Y	3810 ± 1310		Y	3720 ± 1100	
	PD	N	4270 ± 2350	0.981	N	4590 ± 2850	0.305
		Y	4290 ± 3040		Y	3640 ± 2190	
bFGF	pre	N	1.5 ± 1.5	0.591	N	1.5 ± 1.5	0.798
		Y	1.5 ± 2.0		Y	1.5 ± 2.0	
	day 21	N	2.0 ± 2.5	0.900	N	2.5 ± 3.0	0.489
		Y	2.0 ± 4.0		Y	1.5 ± 4.0	
	PD	N	4.0 ± 4.0	0.458	N	4.0 ± 4.5	0.267
		Y	5.0 ± 6.5		Y	6.0 ± 7.5	
PlGF	pre	N	18.0 ± 16.0	0.746	N	19.5 ± 18.0	0.106
		Y	16.5 ± 16.0		Y	12.0 ± 11.0	
	day 21	N	55.5 ± 61.0	0.740	N	58.5 ± 56.5	0.233
		Y	51.0 ± 42.5		Y	40.5 ± 45.0	
	PD	N	19.0 ± 18.0	0.984	N	20.5 ± 19.5	0.519
		Y	19.0 ± 19.0		Y	16.5 ± 14.5	
PDGF-B	pre	N	5040 ± 2680	0.549	N	4980 ± 2600	0.521
		Y	4220 ± 3020		Y	4080 ± 3240	
	day 21	N	3980 ± 2560	0.247	N	4040 ± 2600	0.107
		Y	2600 ± 2260		Y	2120 ± 1780	
	PD	N	3800 ± 3860	0.514	N	4300 ± 3960	0.895
		Y	5000 ± 3800		Y	4560 ± 3760	
IL-8	pre	N	85 ± 90	0.914	N	75 ± 85	0.796
		Y	80 ± 100		Y	90 ± 110	
	day 21	N	105 ± 180	0.339	N	90 ± 165	0.581
		Y	45 ± 50		Y	55 ± 55	
	PD	N	155 ± 165	0.445	N	135 ± 155	0.846
		Y	100 ± 140		Y	120 ± 155	
CCL2	pre	N	318 ± 118	0.646	N	340 ± 120	0.512
		Y	344 ± 126		Y	316 ± 132	
	day 21	N	378 ± 148	0.144	N	412 ± 166	0.767
		Y	426 ± 188		Y	396 ± 190	
	PD	N	330 ± 118	0.822	N	356 ± 180	0.054
		Y	320 ± 194		Y	248 ± 62	
CCL5	pre	N	62700 ± 24200	0.440	N	65800 ± 23200	**0.030**
		Y	58500 ± 24300		Y	50500 ± 23800	
	day 21	N	60200 ± 28500	0.360	N	62300 ± 27000	0.092
		Y	54600 ± 27900		Y	48400 ± 29000	
	PD	N	78200 ± 34800	0.119	N	73600 ± 33900	0.118
		Y	62000 ± 30700		Y	56000 ± 26900	

* DC was achieved in 28 patients indicating as ‘Y’.

** rTS was achieved in 18 patients indicating as ‘Y’.

Pre, pretreatment; DC, disease control; rTS, relative tumor shrinkage; PD, progressive disease; Ang-1, -2, angiopoietin 1, 2; VEGF-A, –C, vascular endothelial growth factor-A, -C; SDF-1, stromal cell-derived factor-1; bFGF, basic fibroblast growth factor; PlGF, placental growth factor; PDGF-B, platelet-derived growth factor; IL-8, interleukin-8; CCL2, 5, chemokine2, 5; SD, standard deviation.

### Change pattern of serum VEGF-A levels and survival

**Figure 3:**
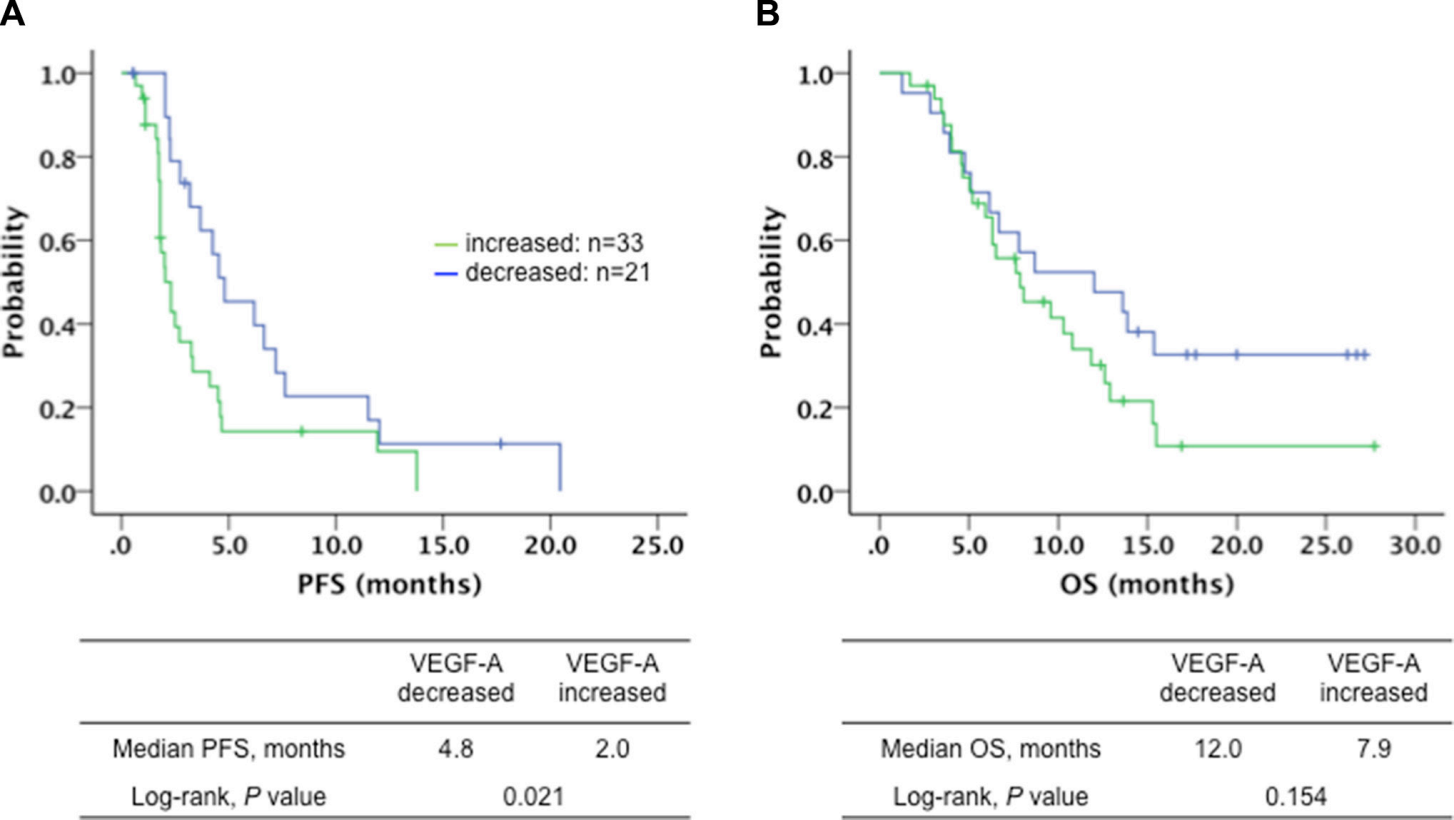
(**A**) Progression-free survival (PFS) (4.8 vs. 2.0 months, Log-rank test, *P* = 0.021) and (**B**) overall survival (OS) (12.0 vs. 7.9 months, Log-rank test, *P* = 0.154) in patients with metastatic colorectal cancer treated with regorafenib according to the serum VEGF-A levels decreased (—, *n* = 33) or increased (—, *n* = 21) after 21 days of treatment.

We further investigated the association of changes in the serum levels of all cytokines before and during treatment point (day 21 after treatment start, and at the time of confirmed progressive disease) with outcome. We observed significantly better PFS when VEGF-A levels decreased after 21 days of treatment compared with when it increased (4.8 vs. 2.0 months, *P* = 0.021) (Figure 3A). There was also a trend toward better OS in same patterns (12.0 vs. 7.9 months, *P* = 0.154) (Figure 3B). VEGF-A levels decreased 21 days after the treatment start followed by an increase in progressive disease (PD) that was associated with improving PFS (4.8 vs. 1.8 months, *P* < 0.001) and OS (12.0 vs. 9.6 months, *P* = 0.104) compared with VEGF-A that increased on day 21 followed by a decrease in PD (Supplementary Figure S1). Correlation between the change pattern and DC or rTS was also investigated. Either VEGF-A decreased (*P* = 0.033) or Ang-2 decreased (*P* = 0.046) after 21 days were significantly associated with achieving rTS. In addition, DC was significantly frequent in patients with VEGF-A levels that decreased after 21 days (*P* = 0.036). There was no significant difference in other cytokines for DC, rTS, PFS or OS at baseline.

### Serum cytokine levels at each test point and toxicity

Fifty-four patients were eligible for safety analysis (Supplementary Table S1). Hand-foot skin reaction (HFSR), hypertension (HT), liver dysfunction checked by AST (aspartate aminotransferase) and ALT (alanine aminotransferase) values, hyperbilirubinemia as total bilirubin (T-BIL) increased, and thrombocytopenia as regorafenib-specific adverse events were selected for analyzing the relationship between measured cytokines levels at baseline and early phase of treatment start. There was no treatment-related death.

Serum Ang-2, bFGF, CCL2 levels at baseline were associated with frequency of adverse events: high Ang-2 levels were associated with T-BIL increase ≥ grade 3 (*P* = 0.010); high bFGF levels were associated with AST (*P* = 0.004) or ALT (*P* = 0.012) increase ≥ grade 3; low bFGF levels were associated with HT ≥ grade 3 (*P* = 0.008) and low CCL2 levels were associated with HFSR ≥ grade 2 (*P* = 0.030) (Table 3). Correlations between serum CCL5 levels divided into two groups by the cut-off (59959 pg/ml) for rTS, and adverse events were also investigated. Serum CCL5 levels ≤ cut-off were associated with any grade of HFSR (Odds ratio 9.1, *P* = 0.025), and of thrombocytopenia (Odds ratio 4.156, *P* = 0.013), and a trend toward T-BIL increase ≥ grade 2 (Table 3).

**Table 3:** Adverse events and serum cytokine levels at baseline (*n* = 54)

Cytokines	AEs	CTCAE Grade	Mean ± SD, (pg/ml)	*P* value
Ang-2	T-BIL increased	< 3	2730 ± 1710	0.010
		≥ 3	5475 ± 2375	
bFGF	AST increased	< 3	1.3 ± 1.6	0.004
		≥ 3	3.6 ± 1.75	
bFGF	ALT increased	< 3	1.35 ± 1.65	0.012
		≥ 3	3.6 ± 2.05	
bFGF	Hypertension	< 3	1.65 ± 1.8	0.008
		≥ 3	0.6 ± 0.6	
CCL2	HFSR	< 2	372 ± 116	0.030
		≥ 2	300 ± 116	

Cytokines	AEs	CTCAE Grade	Odds ratio (95% CI)	*P* value
CCL5 ≤ cut-off	HFSR	0 vs. 1 ≤	9.1 (1.03–80.1)	0.025
CCL5 ≤ cut-off	PLT decreased	0 vs. 1 ≤	4.2 (1.3–13.2)	0.013
CCL5 ≤ cut-off	T-BIL increased	< 2 vs. 2 ≤	3.1 (0.98–9.7)	0.051

AEs, adverse events; CTCAE, the Common Terminology Criteria for Adverse Events; Ang-2, angiopoietin 2; bFGF, basic fibroblast growth factor; CCL2, chemokine 2; T-BIL, total bilirubin; AST, aspartate aminotransferase; ALT, alanine aminotransferase; HFSR, Hand-foot skin reaction; Gr, grade; SD, standard deviation; CCL5, chemokine 5; PLT, platelet.

## DISCUSSION

In our exploratory study, serum levels of CCL5 and VEGF-A were revealed as candidate surrogate markers for efficacy in regorafenib monotherapy. In addition, regorafenib-related toxicities such as HFSR, hyperbilirubinemia, and thrombocytopenia also correlated with CCL5. These adverse events were observed to be highly frequent in the Japanese population in the CORRECT trial [20]. To our knowledge, this was the first exploratory study that investigated the serum cytokines levels at the planned points through treatment from baseline until PD for the novel multi-kinase inhibitor agent regorafenib. Our study is limited by its small sample size and restricted cytokines for testing, especially inflammatory factors including CCL2 and CCL5. Further research with large patient cohorts is warranted to confirm our results by testing comprehensive cytokines in a regorafenib-related pathway.

We focused on finding predictive markers for efficacy and adverse events before treatment or early phase after treatment start. The phase I dose-escalation study (BAY73-4506) showed that plasma VEGF concentration increased over 21 days of multiple dosing of regorafenib and returned to baseline levels during the 7 day treatment break. Conversely, plasma soluble VEGFR2 concentration decreased dose-dependently in each cycle [23]. Therefore, we set up the second blood collection point after baseline on day 21 in our study. The phase I study also revealed a high inhibition of tumor growth at the end of a 21-day dosing period with regorafenib for renal cell carcinoma cell lines characterized by increased synthesis of VEGF [24]. Thus, measurement on day 21 was considered to be the most reasonable point to predict early tumor response or outcome, in addition to baseline.

Recently, some studies reported the correlation between tumor-related markers in blood association with treatment efficacy by chemotherapy [25, 26]. Hayashi et al. demonstrated that an early increase in the serum VEGF-A concentration after the initial decrease is a potential predictive marker of poor tumor response in the second-line FOLFIRI plus bevacizumab for metastatic CRC patients [26]. However, a different treatment-line setting and a blood collection point should be discussed, i.e. our study set up to measure the serum cytokine levels until confirmed PD. According to a previous study investigating association between plasma levels of candidate angiogenic factors with outcome of bevacizumab-combined chemotherapy, variant of VEGF levels during treatment was found to reflect more definitely the anti-cancer situation than baseline [27]. The mechanism of remarkable VEGF-A decrease in good outcome population due to regorafenib dosing has not been cleared till now. As mentioned above, plasma soluble VEGFR2 concentrations decreased after starting treatment with regorafenib, thereby tumor cells induce production of its ligand VEGF-A for their survival or progression [23]. However, there was no evidence of an increased production of VEGF-C as another VEGFR2-ligand, which suggested the production of VEGF-A was stimulated by a different mechanism independent of inhibiting VEGFR2. Furthermore, change patterns of serum Ang-2 levels were similar to VEGF-A in our study, though no significant difference in PFS unlike VEGF-A. Taken together, VEGF-A and Ang-2 might activate an angiogenic mechanism responding to regorafenib, and the presence of a genetically specific mechanism is speculated in patients with a better clinical outcome leading to suppression of angiogenic factor production. Further analysis of genetic variants for angiogenic factors is warranted.

We also focused on chemokine as inflammatory markers, of which CCL5 was determined as with a predictive marker at baseline for efficacy as well as with CCL2 for HFS. We selected the chemokine that might act as not only chemoattactant but also angiogenic factor according to previous reports [28–31].

In a complex chemokine network in human cancers, CCL2 localizes to epithelial areas of the tumor, and CCL5 localizes with tumor-infiltrating leukocytes [29]. Cambien et al. pointed out the ability of CCL5 to promote metastatic features of tumor cells and to diminish anti-tumor immunity [30]. Wang et al. found that CCL5 and CCR5 interaction activates protein kinase Cδ (PKCδ), c-Src and hypoxia-inducible factor-1α (HIF-1α) pathways, particularly leading to upregulation of VEGF expression in osteosarcoma microenvironment [31]. CCL5 thereby is thought to participate in upregulation of VEGF production. Our results demonstrated that the lower serum CCL5 levels were associated with rTS and better PFS and OS. For this reason, we speculated that VEGF production was not stimulated under low CCL5 concentration leading to suppress tumor progression as well as inactivate VEGFR2 signaling after regorafenib dosing. Hence, we proposed that CCL5 and VEGF-A might serve as predictive markers for efficacy at pretreatment and early phase after treatment start.

There have been few reports about regorafenib-related adverse events by measurement of blood samples. In our study, pretreatment serum concentration of Ang-2 and bFGF as angiogenic factors show significant relationship with occurrence of the hepatobiliary adverse events or hypertension, which may represent regorafenib-targeting VEGFR1-3. Hypertension is a well-known cardiovascular event during anti-angiogenic treatment [32]. A recent report revealed that decreased serum levels of bFGF as physiological pro-angiogenic mediator was associated with evidence of hypertension. By contrast, elevated levels of CRP, VEGF and IL-8 as representative of inflammation correlated with hypertension [33]. According to a biomarker study of sorafenib for advanced hepatocellular carcinoma, higher plasma levels of Ang-2 at baseline were significantly associated with higher total bilirubin levels that might explain a mechanism of hepatobiliary dysfunction due to anti-angiogenetic treatment [34]. In another pathway, lower serum CCL2 and CCL5 levels at baseline were associated with HFSR, which is the most significant dermatologic toxicity in regorafenib therapy. Sorafenib and sunitinib overlap with regorafenib in targeting VEGFR 2 and 3, PDGFR and KIT. However, combination of these receptors or the related-pathways was considered to stimulate HFSR development [35]. CCL2 is known to promote M2 phenotype of tumor-associated macrophage polarization leading to angiogenic activation through VEGF-A production [36]. In addition, chemokine production alone is not considered to directly induce keratinocyte apoptosis as well as critical inflammatory state of HFSR [37]. bFGF Taken together with positive interaction between CCL5 and VEGF-A production [30], CCL2 and CCL5 thereby might participate HFSR development through inhibiting angiogenesis pathways.

According to the analysis of association between adverse events and clinical outcome, any grade thrombocytopenia was associated with longer PFS and OS; any grade HFS or HT were associated with longer OS. However, higher grade of these adverse events did not remain significant in outcome (data not shown). This means that CCL5 could be a valid predictive marker of efficacy and toxicity, especially in HFSR at baseline. Further study is needed to disclose the background of ethnic differences in toxicity between Japanese and non-Japanese populations. In addition, our study results and suggestions should be applied to other cancer types such as gastrointestinal stromal tumor, for which regorafenib has also been approved in several countries including US, EU and Japan.

In conclusion, serum CCL-5 and VEGF-A are pretreatment or intra-treatment predictive markers for efficacy and safety for mCRC patients receiving regorafenib in salvage-line setting.

## MATERIALS AND METHODS

### Study design

This exploratory study was performed at a single center in Japan. The safety and efficacy of regorafenib as salvage-line treatment in mCRC patients were evaluated. The aim of this study was to investigate pre-treatment and changes during treatment in serum candidate cytokines as potential markers of treatment response to regorafenib. Our study was designed as an exploratory study but not as a prospective clinical trial, meaning that no intervention was applied to patient treatment. Because of the absence of previous reports for the candidate cytokines in efficacy or safety for regorafenib, no formal statistical assumption was adapted to this study. Therefore, we assumed the required sample size considering both the expected patient enrollments per year at our institute and results of CORRECT study. On the basis of the results in the CORRECT study, the expected disease control rate (DCR) was assumed to be 40% given the refractory mCRC patients being treated with regorafenib. Although the DCR in patients assigned regorafenib and placebo were 40% and 15% in CORRECT study, the objective response rates were 1.0% and 0.4% assigned placebo, respectively. Therefore we estimated that 44 patients would be required for the study to achieve an expected disease control rate of 40%, with a lower limit of 20%, a one-sided α-level of 0.05%, and a power of 90%. To account for exclusions from analysis, we decided that a total of 50 patients would be enrolled within two years. The institutional review board at the Cancer Institute Hospital of the Japanese Foundation of Cancer Research approved the protocol.

### Patients

Eligible patients had a histologically confirmed diagnosis of mCRC; Eastern Cooperative Oncology Group performance status (ECOG PS) of 0–1; history of previous standard chemotherapy consisting 5-FU, L-OHP, CPT-11, bevacizumab, cetuximab or panitumumab; measurable or evaluable disease; life expectancy ≥ 12 weeks, and signed informed consent. Patients with any of the following conditions were excluded: active infection; interstitial lung disease, severe emphysema or pulmonary fibrosis; paralytic or mechanical bowel obstruction; uncontrolled hypertension; uncontrolled diabetes; cirrhosis; clinically significant cardiovascular disease; history of myocardial infarction within the previous 3 months; uncontrolled angina pectoris or arrhythmia; multiple primary cancers within the past 5 years; clinically significant mental or psychological disease; any other condition making a patient unsuitable for this study.

All patients initially received 160 mg regorafenib (Bayer, Leverkusen, Germany) once daily for the first three weeks of each 4-week cycle. Doses were adjusted on the basis of haematological and non-haematological adverse events by treating physician's discretion in accordance with the manufacturer's recommendations.

### Evaluation of safety and efficacy

Data on the patients, including the results of imaging studies, were recorded in electronic clinical records. A multidisciplinary hospital colorectal cancer team confirmed patient eligibility. In all patients, adverse events (AEs) were graded according to the Common Terminology Criteria for Adverse Events (CTCAE), version 4.0 every week during the first two cycles and every two weeks or before each treatment cycle after the third cycle. Treatment was continued until any of the following occurred: disease progression, unmanageable toxicity, patient refusal, or transfer of the patient to another hospital. The baseline tumor response was assessed within 4 weeks before enrollment in the study, and the tumor response was then assessed prospectively every 8 weeks by computed tomography according to the Response Evaluation Criteria for Solid Tumors (RECIST), version 1.1.

### Sample collection and analysis method

Blood samples were obtained from all enrolled patients at baseline before the first dose of regorafenib, as well as, day 21 after treatment start, and at the time of confirmed progressive disease (PD). Serum was separated from blood sample and stored at −80°C at our institute. The serum levels of the 11 cytokines; angiopoietin-1 (Ang-1), angiopoietin-2 (Ang-2), vascular endothelial growth factor-A (VEGF-A), vascular endothelial growth factor-C (VEGF-C), stromal cell-derived factor-1 (SDF-1), platelet-derived growth factor beta (PDGF-β), placental growth factor (PlGF), basic fibroblast growth factor (bFGF), interleukin-8 (IL-8), chemokine (C-C motif) ligand 2 (CCL2), chemokine (C-C motif) ligand 5 (CCL5) were measured using Quantikine ELISA kits (R&D Systems) according to the manufacturer's instructions. In brief, we added 100 μL of samples to 96 well plates coated with antibody to each cytokine and incubated for two hours. After washing with wash buffer, we then added 200 μL of horseradish peroxidase-conjugated antibody to each cytokine and incubated for one or two hours. After further washing with wash buffer, substrate solution was added and the absorbance of the samples at 450 nm as well as 570 nm were measured using xMark microplate spectrophotometer (BioRAD). The samples were measured in triplicate and the average values were used for further analysis.

### Statistical analysis

The disease control rate (DCR) was calculated from the number of patients who achieved a complete response (CR), partial response (PR), or stable disease (SD) with treatment, while the objective response rate (ORR) was based on the number of patients who had CR or PR. To distinguish tumor shrinkage (TS) or early tumor shrinkage (ETS) generally analyzed in first-line treatment, we used relative tumor shrinkage (rTS) defined as a relative change of ≥ 0% in the sum of the longest diameters of target lesions at the first evaluation when compared with baseline. Enrolled patients were divided into two subgroups as disease control (DC) and Non-DC as well as rTS and Non-rTS for analysis. Baseline serum cytokine levels were compared between these subgroups, and correlation between changes in each serum cytokine levels and efficacy including DC or rTS, progression-free survival (PFS) and OS were also evaluated. In addition, adverse events were compared with outcomes and change of cytokine levels. The chi-square test for independence (Fisher' s exact test when the expected value was < 5) was employed to compare the incidence of adverse events for the tested serum cytokines levels. The changes were evaluated between two of the three points (baseline, day 21, PD) of collecting samples, and its patterns were defined as ‘increased’ or ‘decreased’ from earlier collected points. Differences in the means of continuous measurements between two points were tested by the Student's unpaired *t* test. One-way analysis of variance (ANOVA) was used to evaluate differences between three measurement points: baseline, day 21 and PD. To seek the optimal cut-off point that predicts DC or rTS at baseline, the receiver operating characteristic curve (ROC) analysis was analyzed. The cutoff was decided as the point on the ROC curve with the largest average sensitivity and specificity. Subgroups divided by using the cutoff were compared for tumor response, PFS and OS. PFS was defined as the interval between the date of starting treatment and the date of confirming disease progression or death. Data of patients without disease progression were censored on the date at which the patient was last confirmed to be alive. OS was calculated from the date of starting treatment until the date of death from any cause. In patients who were lost to follow-up, data were censored on the date when the patient was last confirmed to be alive. The median follow-up time for survival was calculated by means of the reverse Kaplan–Meier method. PFS and OS were estimated by the Kaplan-Meier method and were compared using the log-rank test, with predictive or prognostic factors being identified by univariate analysis. Multivariate analysis of the factors was conducted by using the Cox proportional hazards model to identify factors influencing PFS and OS. All analyses were carried out with SPSS software, version 22.0 (IBM Corporation, Armonk, NY, USA) and *P* < 0.05 was considered to indicate statistical significance.

## SUPPLEMENTARY MATERIALS FIGURES AND TABLES


