# Supplementary material for: Serum VEGF-A and CCL5 levels as candidate biomarkers for efficacy and toxicity of regorafenib in patients with metastatic colorectal cancer
Source: Oncotarget. 2016 May 5;7(23):34811–23. doi: 10.18632/oncotarget.9187 (PMC5085191; doi:10.18632/oncotarget.9187)
Supplement: Supplementary file 1 [file oncotarget-07-34811-s001.pdf]

Serum VEGF-A and CCL5 levels as candidate biomarkers for efficacy and toxicity of regorafenib in patients with metastatic colorectal cancer

Supplementary Materials

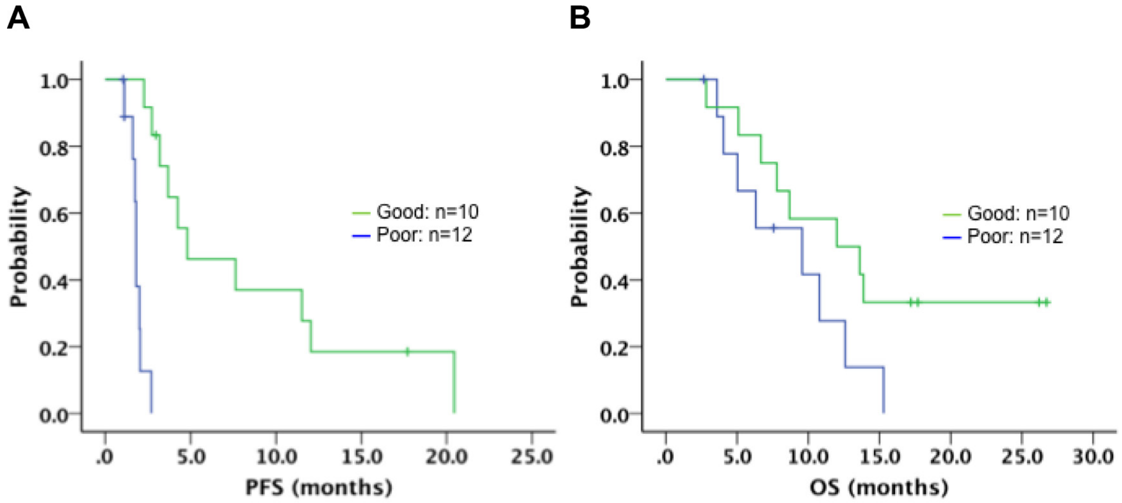

	Good: VEGF-A Dec followed by Inc	Poor: VEGF-A Inc followed by Dec
Median PFS, months	4.8	1.8
Log-rank, *P* value	*P*<0.001	

	Good: VEGF-A Dec followed by Inc	Poor: VEGF-A Inc followed by Dec
Median OS, months	12.0	9.6
Log-rank, *P* value	*P*=0.104	

Supplementary Figure S1: (**A**) Progression-free survival (PFS) (4.8 vs. 1.8 months, Log-rank; $P < 0.001$) and (**B**) overall survival (OS) (12.0 vs. 9.8 months, Log-rank test, $P = 0.104$) in patients with metastatic colorectal cancer treated with regorafenib according to VEGF-A levels decreased (Dec) on day 21 followed by increased (Inc) at progressive disease (—Good, $n = 10$) or the opposite (—Poor, $n = 12$).

Supplementary Table S1: Adverse events according to CTACE grade ($n = 54$)

Adverse events	Gr1	Gr2	Gr3	Gr4	Gr ≥ 3
					***N* (%)**
HFSR	14 (25.9)	14 (25.9)	18 (33.3)	0	18 (33.3)
Hypertension	0	10 (18.5)	6 (11.1)	0	6 (11.1)
AST increased	17 (31.5)	5 (9.3)	4 (7.4)	1 (1.9)	5 (9.3)
ALT increased	14 (25.9)	4 (7.4)	3 (5.6)	1 (1.9)	4 (7.4)
T-BIL increased	18 (33.3)	18 (33.3)	3 (5.6)	0	3 (5.6)
Thrombocytopenia	16 (29.6)	5 (9.3)	1 (1.9)	1 (1.9)	2 (3.7)

CTCAE, the Common Terminology Criteria for Adverse Events; Gr, grade; HFSR, Hand-foot skin reaction; AST, aspartate aminotransferase; ALT, alanine aminotransferase; T-BIL, total bilirubin.
